# An Ultrasonic Object Detection Applying the ID Based on Spread Spectrum Technique for a Vehicle †

**DOI:** 10.3390/s20020414

**Published:** 2020-01-11

**Authors:** Donghee Yi, Heetae Jin, Moon Chan Kim, Suk Chan Kim

**Affiliations:** 1 Department of Electrical and Computer Engineering, Pusan National University, 2, 63beon-gil, Busandaehakro, Geumjeong-Gu, Busan 46241, Korea; ldh2717@pusan.ac.kr (D.Y.); htjin@pusan.ac.kr (H.J.); 2 Department of Naval Architecture and Ocean Engineering, Pusan National University, 2, 63beon-gil, Busandaehakro, Geumjeong-Gu, Busan 46241, Korea; kmcprop@pusan.ac.kr

**Keywords:** ultrasound, detection, DSSS, orthogonal code, DQPSK

## Abstract

When an ultrasonic sensor generates an ultrasonic wave and detects an obstacle from a reflected wave, a signal transmitted by other ultrasonic sensors would be interference. In this paper, to overcome the interference, a transducer transmits a signal with a unique ID modulated. The interference is ignored by verifying that the reflected signal includes its ID. The ID verification process uses a correlation between the received signal and the ID. Therefore, the ID is selected from orthogonal codes with good cross-correlation. Long code has the advantage of being more robust to interference. However, the reflected wave from nearby obstacles might return before the transmission ends. Therefore, the 7-bit Barker code is applied for near obstacle detection and a 31-bit Gold code is used for distant obstacle detection. The modulation technique is DQPSK, which is available in a narrow bandwidth and has a simple receiver structure. In ID recognition based on correlation, a near–far problem occurs due to a large amplitude difference between the received wave and interference. The addition of a zero-crossing detector solves this problem. The hardware is implemented based on the algorithm proposed in this paper. The simulation showed a detection rate of at least 90% and the the result of the real measurement represented a detection rate of 97.3% at 0.5 m and 94.5% at 2 m.

## 1. Introduction

Self-driving cars are the next-generation car that aims to drive safely by perceiving the driving environment and setting the route without the control of the driver automatically. Since fully automatic driving is not possible yet, a system to assist a driver has been developed. An advanced driver assistant system (ADAS) warns the driver when a dangerous situation happens. It includes systems such as lane keeping assistance system (LKAS), autonomous emergency braking system (AEB), forward collision warning system (FCWS), and adaptive cruise control (ACC). Among them, AEB and FCWS use ultrasonic sensors to recognize near objects through ranging sensors using light detection and ranging (LiDAR), radars and cameras [1] are used to observe around the car more accurately [2]. Because those sensors, except for the camera, emit a very short pulse, the detection within 1 m needs data collection and processing time within nanoseconds. Camera sensors cannot determine the distance from the obstacle. So, the combination of those sensors cannot detect close objects. For example, the parking assistance system (PAS) prioritizes the detection of short-range objects within 1 m and sometimes uses ultrasonic sensors for detection [3]. A current ultrasonic module for vehicles calculates the distance to an object by measuring the round trip time of a transmitted pulse. However, if the ultrasonic module equipped to adjacent vehicles operates simultaneously in the same space, the pulse from other sensors become interference, which results in a reflected wave not being detected correctly. To solve this problem, time division multiple access (TDMA) could be considered, which divides the operation time of each sensor evenly to allow only one sensor operating in a given slot. However, the sensors of all vehicles in the same space cannot be controlled at the same time, and it is impossible to distribute the time slots without overlapping. Instead, frequency division multiple access (FDMA), where each sensor transmits and receives with a different frequency, can overcome interference between sensors without simultaneous control. However, sensor manufacturers cannot provide the transducers with different resonance frequencies and wide bandwidth because of the cost. As a result, code division multiple access (CDMA) can be applied without considering time and frequency resource allocation. This technique uses an orthogonal code to identify the transmitter. Orthogonal codes have a strong correlation only with the same code. If the transmitter has the ID in the form of orthogonal code, it distinguishes the reflected wave, including its ID, by correlation. Using the properties of this technique, the simulation was conducted in [4,5] and the implementation of a vehicle ultrasonic sensing system that can overcome interference and detect obstacles are introduced in this paper. The rest of the paper is organized as follows. Section 2 shows research on object detection. Section 3 introduces the hardware configuration, modulation, ID selection, and implementation of object detection. Section 4 shows the result of the algorithm with simulation and testing with the implemented hardware. Section 5 summarizes the results of Section 4, and Section 6 addresses future research.

## 2. Related Work

In [6,7], the time-of-flight (ToF) method is used to measure the distance from obstacles using ultrasonic waves in an indoor localization system. This method focuses on the accuracy of distance measurement. Thus, each sensor cannot distinguish the reflected wave from others. M. O. Khyam et al. [8] applied a chirp signal to ultrasonic ranging. However, there is only one chirp pattern, and it cannot be used for indoor ranging systems that need to distinguish multiple sensors. A. Ens and L. M. Reindl [9] applied orthogonal frequency division multiplexing (OFDM) to the ranging system used for localization. This scheme uses multiple subcarrier for transmission, improving the data transmission efficiency and reducing the length of the transmitted wave. However, orthogonal frequency division multiple access (OFDMA) is not applied because subcarriers on the transducer in [9] are too narrow to divide into multiple groups.

The automotive ultrasonic sensor has a low frequency and a narrow bandwidth, and the signal processing algorithm of the sensor for vehicles aims for accurate ranging. Therefore, algorithms for vehicles have a constraint to the application of signal processing techniques, which include multiple access schemes such as CDMA. S. Shin et al. [10] measure the distance with pulse interval modulation. Also, the author distinguishes each pulse by increasing the transmission rate and applying a gradually increasing pulse interval. However, it is not known whether the interference can be overcome since interference is not applied in the test. In [11], the authors use the ultrasonic sensor to measure distance and a planar electrode to get capacitance. The combination of the sensor and the electrode improves the distance accuracy, and the calculated distance is continuously passed through the Kalman filter to compensate for false detection due to interference. However, objects that suddenly appear at close range might not be detected due to the smoothing effect of the filter. On the other hand, the ultrasonic sensors used in indoor positioning have a wide choice of resonant frequency and bandwidth. Therefore, the limited applications of multiple access schemes have been researched.

L. Segers et al. [12] applied CDMA to an indoor localization system. CDMA is divided into direct sequence spread spectrum (DSSS) encoding signal based on a spread spectrum technique and frequency hopping spread spectrum (FHSS) encoding frequency variation pattern. The authors compared the performance of ranging systems to implement each of the two spread spectrum techniques. The results show that DSSS is more accurate than FHSS, and FHSS is more robust to interference than DSSS. However, FSSS and is only available with a wideband transducer, and the DSSS has the constraint in length of the orthogonal code on the short-range measurement. Also, the transmitter always has a synchronization wire connected to the receiver. Therefore, it has a disadvantage that the system applies coherent detection only. In [13], the authors implemented a system for indoor ranging using CDMA based on DSSS. The system modulated the code sequence with FSK. Implementing FSK as a narrowband transducer limits the transmission rate. As a result, a long wave for transmission is generated, which makes it impossible to detect a round trip time at a short distance. Conversely, reducing the length of the wave as the code length decreases results in poor cross correlation. In [14,15], chirp spread spectrum (CSS) was selected as the multiple access scheme. Multiple sensors distinguish each other using various chirp patterns as an identifier. However, CSS requires fast phase shifts, and their implementation is available only on a wideband transducer.

## 3. System Description

### 3.1. Modulation

In order to transmit an ID, a modulation scheme should be determined. Frequency shift keying (FSK), which converts information into frequency components, has the advantage of simplicity because the demodulator does not include a synchronization circuit. However, as modulation order of symbol increases, the number of modulated frequency increases, and bandwidth becomes wider. A transducer for automotives have a narrow bandwidth and are inadequate for FSK. Another technique is phase shift keying (PSK), which transforms data into the phase of carrier wave with a constant frequency [16]. Since all symbols are modulated with the same frequency, the bandwidth does not increase when the number of phases increases. However, a receiver needs a synchronization circuit such as a phase locked loop (PLL), and the complexity of the receiver is increased. As an alternative, the system uses a differential phase shift keying (DPSK) that modulates the data into a phase difference between two consecutive symbols [17,18]. This modulation can be implemented with incoherent detection as well as coherent detection. In the coherent detection, the demodulator multiplies the received wave with the sinusoidal wave with a reference frequency. On the other hand, the demodulator using the incoherent detection multiplies received wave with the one delayed by one symbol. This does not require the generation of the sinusoidal wave with a reference frequency and phase. Therefore there is no PLL in the circuit. Therefore, this type of receiver has the advantage of lower complexity and cost despite a narrow bandwidth of the transducer. The modulation order of the DPSK is related to the length of the transmitted wave. As the modulation order increases, the number of bits represented by a symbol increases, so the length of the wave is shortened. Therefore, the ultrasonic ranging system can measure short round trip time and detect near obstacles. This system uses $\frac{\pi }{4}$-DQPSK to reduce the length of the wave. In this modulation, two bits {$a_{k},b_{k}$} of k-th symbol select one of four differential phases.

(1) $$\Delta \phi_{k}=\pm \frac{\pi }{4},\pm \frac{3\pi }{4}$$

It is then added to the phase of the previous symbol. When the phase of (*k* − 1)-th symbol $\phi_{k-1}$ given, the phase of *k*-th symbol is

(2) $$\phi_{k}=\phi_{k-1}+\Delta \phi_{k}$$

When *S* is the power of the transmitted wave $s_{k}\left[n\right]$ representing k-th symbol is expressed by

(3) $$\begin{array}{cc} s_{k}\left[n\right] & =\sqrt{2S}\sin\left[\omega n+\phi_{k}\right]\;p\left[n-kN_{sym}\right]\\ & =\sqrt{2S}\sin\left[\omega n+\phi_{k-1}+\Delta \phi_{k}\right]\;p\left[n-kN_{sym}\right] \end{array}$$

where ω is the carrier frequency, $N_{sym}$ is the number of samples per symbol and p[n] is

(4) $$p\left[n\right]=\left\{\begin{array}{cc} 1, & 0\leq n<N_{sym}\\ 0, & \mathrm{otherwise} \end{array}\right.$$

Therefore, the transmitted wave of all the symbol is

(5) $$\begin{array}{cc} s[n] & =\sum_{k=0}^{k=\infty }s_{k}\left[n\right]\\ & =\sum_{k=0}^{k=\infty }\sqrt{2S}\sin\left[\omega n+\phi_{k}\right]p\left[n-kN_{sym}\right]. \end{array}$$

The transmitted wave is attenuated by reflection on the object and reaches the transducer. The transducer has the same effect as a bandpass filter. Assuming that *A* is the attenuation coefficient according to the round trip distance of the transmitted wave, the received wave at the transducer is

(6) $$\begin{array}{cc} r[n] & =A\cdot s[n]\\ \; & =\sum_{k=0}^{k=\infty }A\;\sqrt{2S}\sin\left[\omega n+\phi_{k}\right]\;p\left[n-kN_{sym}\right] \end{array}$$

Delay and noise of the filter output are not considered in Equation (6). When it is assumed that $m_{I}\left[n\right]$ and $m_{Q}\left[n\right]$ are multiplier outputs of in-phase and quadrature channels respectively, outputs are

(7) $$\begin{array}{cc} m_{I}\left[n\right] & =r\left[n\right]\;r\left[n-kN_{sym}\right]\\ \; & =2A^{2}S\sum_{k=0}^{k=\infty }\sin\left[\omega n+\phi_{k}\right]\sin\left[\omega \;n+\phi_{k-1}\right]\\ \; & \;\;\;\;\;\;\;p[n-kN_{sym}]\\ \; & =A^{2}S\sum_{k=0}^{k=\infty }(\cos\left[\phi_{k}-\phi_{k-1}\right]\\ \; & \;-\cos\left[2\omega n+\phi_{k}+\phi_{k-1}\right])\;p\left[n-kN_{sym}\right] \end{array}$$

and

(8) $$\begin{array}{cc} m_{Q}\left[n\right] & =P\left(r_{k}\left[n\right]\right)\;r_{k}\left[n-kN_{sym}\right]\\ \; & =2A^{2}S\sum_{k=0}^{k=\infty }\cos\left[\omega n+\phi_{k}\right]\sin\left[\omega n+\phi_{k-1}\right]\\ \; & \;\;\;\;\;\;\;p[n-kN_{sym}]\\ \; & =A^{2}S\sum_{k=0}^{k=\infty }(\sin\left[\phi_{k}-\phi_{k-1}\right]\\ \; & \;+\cos\left[2\omega n+\phi_{k}+\phi_{k-1}\right])\;p\left[n-kN_{sym}\right] \end{array}$$

where P(r[n]) is $\frac{\pi }{2}$-shift filter using hilbert transform. Then each output is filtered by a lowpass filter.

(9) $$\begin{array}{cc} y_{I}\left[n\right] & ={\left[m_{I}\left[n\right]\right]}_{LPF}=A^{2}S\cos\Delta \phi_{k}\\ \; & =\left\{\begin{array}{cc} \frac{\sqrt{2}A^{2}S}{2}, & \Delta \phi_{k}=\pm \frac{\pi }{4}\\ -\frac{\sqrt{2}A^{2}S}{2}, & \Delta \phi_{k}=\pm \frac{3\pi }{4} \end{array}\right. \end{array}$$

and

(10) $$\begin{array}{cc} y_{Q}\left[n\right] & ={\left[m_{Q}\left[n\right]\right]}_{LPF}=A^{2}S\sin\Delta \phi_{k}\\ \; & =\left\{\begin{array}{cc} \frac{\sqrt{2}A^{2}S}{2}, & \Delta \phi_{k}=\frac{\pi }{4},\frac{3\pi }{4}\\ -\frac{\sqrt{2}A^{2}S}{2}, & \Delta \phi_{k}=-\frac{\pi }{4},-\frac{3\pi }{4} \end{array}\right. \end{array}$$

### 3.2. Multiple Access Scheme

To overcome the constraint of frequency and time resources, the implementation of the detection algorithm includes CDMA based on DSSS [19]. In this technique, the transmitter encodes a bit stream into orthogonal codes before modulation. The receiver can recover the stream only encoded with the orthogonal code of the transmitter because the orthogonal code has very little cross correlation with different codes. For this reason, if each transducer transmits a different orthogonal code, it can distinguish the reflected wave with its own ID from others.

**Figure 1:**
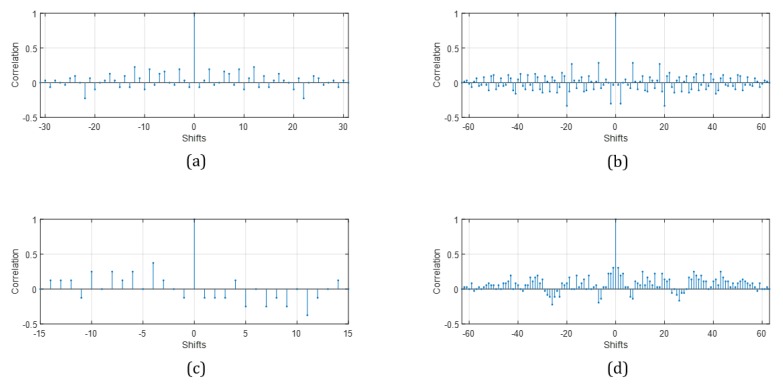
Autocorrelation of orthogonal codes. (**a**) 31-bit Gold code; (**b**) 63-bit Gold code; (**c**) 15-bit Kasami code; (**d**) 63-bit Kasami code.

Gold and Kasami code sequences have good cross-correlation. This property is shown in Figure 1. Four plots in the figure show the low side lobes and high peak at the center of the plot. Both sequences have generation rules. A set of Gold code sequences can be generated if code length *n* is not a multiple of 4, and the size of the sequences is $2^{n}-1$. Kasami code can be generated when *n* is even, and if *n* divided by 4, the size of the code set is $2^{n/2}-1$, otherwise $2^{n}-1$. If the large code set exists, a transducer can choose one of many codes, which increases the probability that the ID of the transducer will not overlap with other IDs. So, the code should be selected from a large set with a good correlation in order to distinguish the reflected wave from interference. Then, Gold code set and the large set of Kasami sequences are selected as the ID. However the large code set has a trade-off. It contains long codes. Therefore, the length of the transmitted wave is also long. If obstacles are near to a transducer, the reflected wave reaches the transducer before the transmission ends. It means that a near object is out of range for detection, and a short-length code has an advantage in short-range detection. For example, the DQPSK modulator in the ultrasound module modulates a 31-bit Gold code into a signal containing 16 symbols. If one symbol contains 12 pulses, there are 192 pulses in the modulated 31-bit signal. Under these conditions, a transducer with a 48 kHz resonant frequency transmits a modulated ID for 400 us and cannot receive it at the same time. During this time, the ultrasound travels 1.36 m at a speed of 340 m/s and takes a 0.68 m round trip. If the obstacle is closer than 0.68 m from the transducer, the reflected wave reaches the ultrasonic sensor before the end of the transmission. Therefore, the theoretical minimum measurable distance is 0.68 m. Besides, the nature of the piezoelectric transducer makes a phenomenon called the ringing. The ringing is a vibration following the end of the electrical excitation, which generates the ultrasonic wave in the sensor. This phenomenon is necessary to exhaust the electrical and mechanical energy remaining after ID transmission. During this time, the transducer cannot receive any signal. The duration of this state is approximately 0.016 s, and the round trip distance during this duration is 0.272 m. The round trip distance for the transmission time and the ringing time is 0.952 m, approximated to 1 m. As a result, the ID should be shorter than 31 bits to measure distances less than 1 m. The 7-bit code is modulated with four symbols under the same conditions as the 31-bit code transmission, and the sensor transmits the modulated signal for 100 us. During this time, an ultrasonic wave at a speed of 340 m/s takes a 0.17 m round trip. The round trip distance during the ringing time is the same as before, so the minimum measurable distance is 0.442 m and approximated to 0.5 m. Therefore, the sensor uses the 31-bit Gold code to measure distance, while the 7-bit code is used additionally for measuring obstacles less than 1 m away.

The round trip distance during the length of the ringing time is the same as before, so the minimum measurable distance is 0.442 m and approximated to 0.5 m. Therefore, the sensor uses the 31-bit Gold code to measure distance, while the 7-bit code is used additionally for measuring obstacles less than 1 m away. The preferred polynomials used as the input of Gold code sequence generator are represented in [20], and the generator in this paper uses two polynomials, which include $x^{5}$+$x^{2}$+1 and $x^{5}$+$x^{4}$+$x^{3}$+$x^{2}$+1 for this implementation. The 7-bit code is selected from the Barker sequences in [21]. It is a sequence consisting of 1 and -1 like Gold code and Kasami code and has the ideal autocorrelation property. However, the code length is much shorter than other codes, and only one code exists for each length [22].

### 3.3. Detection Process

**Figure 2:**
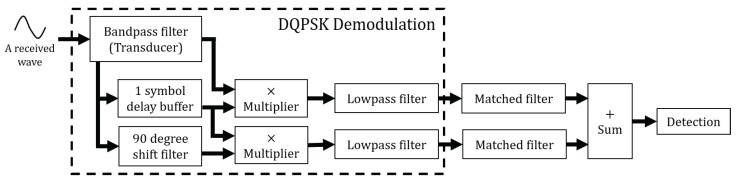
Detection process of the reflected wave.

If the demodulated signal has a strong correlation with the ID of the transducer, the correlation peak indicates when the reflected wave returns to the sensor. Therefore, in the next stage of demodulation, a matched filter is added to obtain the correlation between the demodulated signal and ID. Figure 2 shows the detection process after demodulation. In this figure, two matched filters are connected to the end of demodulator.

The output of the matched filter is obtained by correlating a known delay signal with an unknown input signal. In this algorithm, the known delay signal is the code sequence, and the unknown input signal is the demodulated signal. DQPSK is a modulation scheme that divides data into in-phase and quadrature signals. In the case of a 31-bit Gold code, 16- and 15-bit streams are sent through the two signals, respectively. To get the correlation with the 31-bit Gold code, the outputs from the two matched filters for the 16-bit and 15-bit sequences are stored temporarily. The outputs $R_{I}\left[n\right],R_{Q}\left[n\right]$ are:

(11) $$\begin{array}{c} R_{I}\left[n\right]=y_{I}\left[n\right]*C_{I}\left[N_{seq}-n\right] \end{array}$$

(12) $$\begin{array}{c} R_{Q}\left[n\right]=y_{Q}\left[n\right]*C_{Q}\left[N_{seq}-n\right] \end{array}$$

where $N_{seq}$ is the number of samples of a symbol sequence and $C_{I}\left[n\right],C_{Q}\left[n\right]$ are the upper and lower bits of the ID sequence. The correlation between the 31-bit code and the demodulated signal is expressed by adding the two outputs. This step is also expressed in Figure 2.

(13) $$R\left[n\right]=\frac{R_{I}\left[n\right]+R_{Q}\left[n\right]}{2N_{sym}}$$

Although there is no received wave, the correlation peak might exist. This situation is misdetection. To decrease the misdetection, if the peak is above a threshold, the ultrasonic modem determines to detect its ID. A fixed threshold [23] is determined based on the power of the noise. The value higher than the noise level is set for the threshold. On the other hand, an adaptive threshold [24] that changes repeatedly is suitable for a rapidly changing environment. For example, if the amplitude of the noise changes continuously, misdetection happens frequently. The threshold based on the result of the previous measurement improves detection performance. The detection algorithm in this paper includes the fixed threshold.

### 3.4. Interference from the Other Transducer

**Figure 3:**
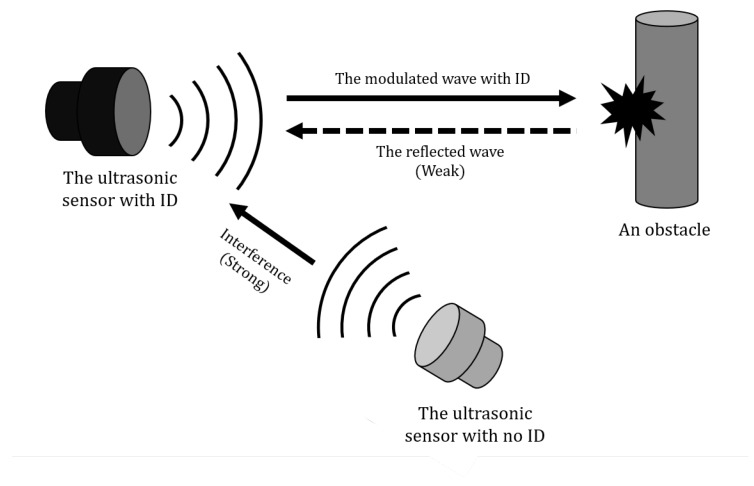
An example of the near–far problem.

CDMA is an efficient multiple access technique in terms of resource usage. However, the near–far problem is known as a weak point. A strong signal of a mobile station located near the base station interferes detection of a weak signal of the mobile station far from the base station. Likewise, if two or more transducers transmit an ID sequence, the reflected wave has a smaller amplitude than the interference as shown in Figure 3. The correlation between the interference and the ideal ID sequence is stronger than the correlation between the demodulated ID and the ideal ID sequence. In this situation, it is a solution to control the signal strength of the transmitter so that the base station receives signals of the same strength from all transmitters. However, a single controller cannot change the sound pressure level of ultrasonic sensors on all the automotive. Therefore, the receiver needs a solution to overcome the interference by additional signal processing and adds a zero-crossing detector before the demodulation step.

**Figure 4:**
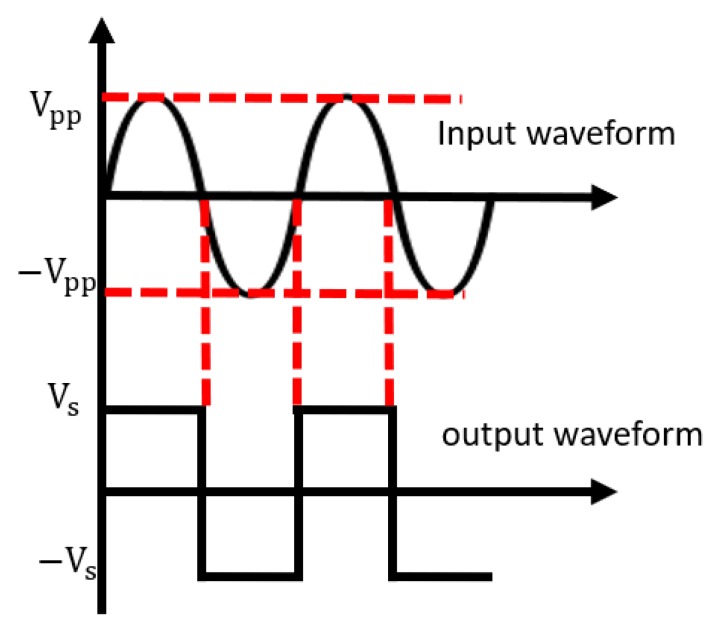
Operation of the zero-crossing detector.

In Figure 4, the zero-crossing detector converts the positive value to $V_{s}$ and the negative value to $-V_{s}$ in the samples of the received wave. $V_{s}$ equals 1 in this algorithm. As a result, the demodulated ID sequence and the interference wave have a similar range of amplitude by scaling. The outputs of zero-crossing detector $r_{I}^{\prime }\left[n\right],r_{Q}^{\prime }\left[n\right]$ are expressed using unit step function u(t).

(14) $$r_{I}^{\prime }\left[n\right]=2u\left(r_{I}\left[n\right]\right])-1$$

(15) $$r_{Q}^{\prime }\left[n\right]=2u\left(r_{Q}\left[n\right]\right)-1$$

**Figure 5:**
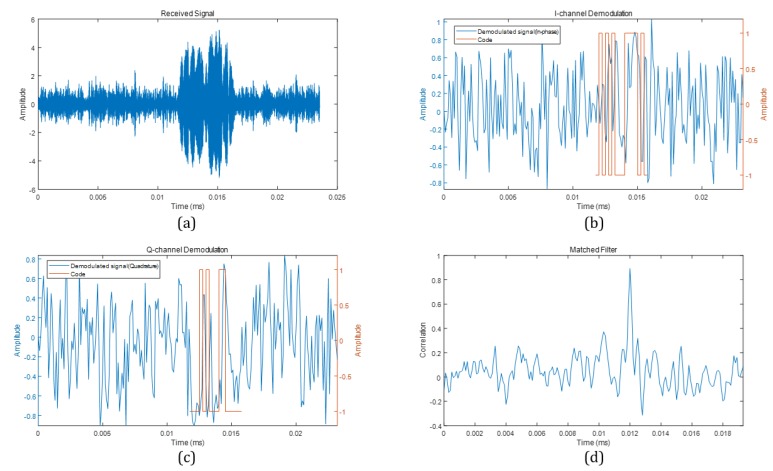
ID detection process. (**a**) Received signal; (**b**) Demodulated signal (In-phase); (**c**) Demodulated signal (Quadrature); (**d**) Matched filter.

Figure 5 shows the demodulation and detection of the reflected wave. Figure 5a represents plot of the received wave. Figure 5b,c represent the demodulated signal by blue line and code sequence by orange line. Figure 5d is the correlation plot between the code sequence and the demodulated signal. The correlation is calculated as in Section 3.3.

## 4. Ranging Implementation

**Figure 6:**
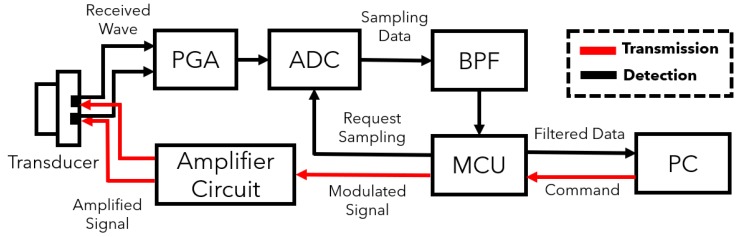
Block diagram of the ultrasonic modem.

Figure 6 shows the structure of the ultrasonic modem. There are two paths in the modem. Black line is used to receive wave, and red line is for transmission. The modem is divided into a transceiver logic and an amplifier part. Transceiver logic consists of MCU and PC, and an amplifier part consists of a programmable gain amplifier (PGA), an ADC chipset, an amplification circuit, and a transducer. PGA and ADC convert the received wave to the digital signal. The bandpass filter reduces the noise level of the received signal. MCU and PC conduct the algorithm in the receiving process and generate the modulated signal in the transmission process. The amplifier circuit is used for amplification of a transmitted signal.

**Figure 7:**
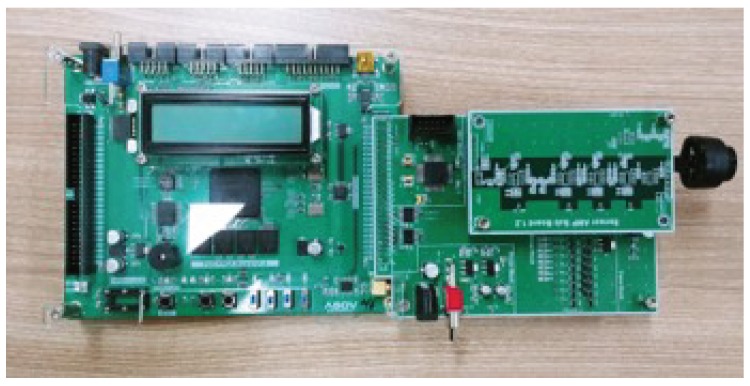
Implemented ultrasonic modem.

**Figure 8:**
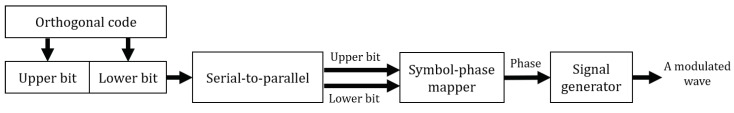
Generation process of the modulated ID.

The implemented hardware is shown in Figure 7. The transducer is front and rear side parking sensors of the Chevrolet Orlando. The sensor has a resonant frequency of 48 kHz and a bandwidth from 46 kHz to 50 kHz. ABOV MC93F5632 MCU, which has a sampling frequency of 1.25 Msps gets the samples of the received wave. ABOV A94Q116, based on the 8051 architecture, controls transmission and receiving on this system. The chipset is an 8-bit MCU with limited computational performance. Therefore, the MCU only triggers the generation of the signal and send the samples of the received wave to PC. Figure 8 shows the generation process of the modulated ID. This process is described in Section 3.1 and can be replaced with the lookup table in the MCU. Demodulation and detection are conducted by Matlab run on PC. The measurement procedure is as follows:

1. The MCU calls the stored code to generate the modulated signal as shown in Figure 8.
2. The signal is amplified through the analog circuit and transmitted from transducer.
3. The transducer receives a reflected wave and ADC chip conducts sampling.
4. The zero-crossing detector converts each sample to 1 or –1.
5. Signal sampled at a previous step is demodulated.
6. The demodulated signal is filtered by a matched filter.
7. Correlation which is the output of the matched filter shows an arrival time of the ID sequence.
8. Distance from an obstacle is calculated using the arrival time.
9. If an error of the calculated distance is less than 10 cm, the ID is detected correctly.

### 4.1. Simulation

The interference is generated in forms of random 7-bit sequence and appears in the receiving window with a 70% probability. The probability is a value considering the propagation time of the ultrasonic transmission command in the module. Additive white Gaussian noise (AWGN) is reflected on the channel of this simulation. The parameters applied to the simulation are shown in Table 1.

**Figure 9:**
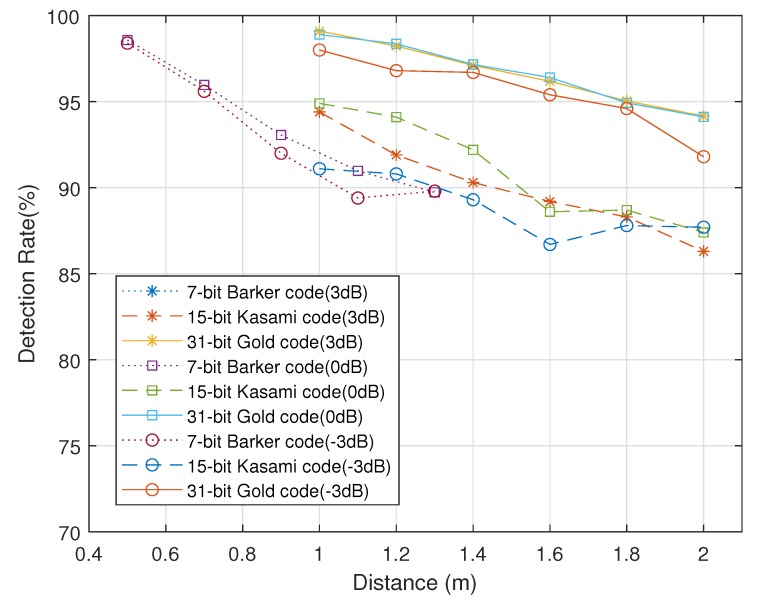
Detection rate on the simulation.

An atmospheric pressure of 101.325 kPa equals 1 atm defined as the standard atmosphere. A temperature of 20 °C is also the standard temperature defined by United States Environmental Protection Agency (EPA). Attenuation coefficient is calculated based on temperature, humidity, atmospheric pressure, and frequency of ultrasound. Given the nonattenuated magnitude $A_{0}$, the magnitude of attenuated signal is expressed as:

(16) $$A=A_{0}\times 10^{\left(-1.46017\times d/10\right)}$$

where *d* is a distance traveled. The simulation was conducted by increasing the distance from the obstacle by 0.2 m. The 7-bit Barker code is used measured from 0.5 to 1.3 m and 31-bit Gold code is used measured from 1 to 2 m. As mentioned in Section 3.2, the ultrasonic modem measures distance greater than 1 m using the 31-bit code. And the 7-bit code is used for distance measurement within 1 m. For this reason, the measurement range is divided into two. The modem measures distances within 2 m due to the limited memory size. This range also applies to the simulation. The measurement at each distance is repeated 1000 times. In Figure 9, the results are expressed as the detection rate, which is the percentage of the measurements with an error less than 10 cm.

When the SNR is increased from −3 dB to 3 dB by 3 dB, there is no significant change in the detection rate and it decreases as distance increases with both codes. Also, the results of 7-bit Barker code show a detection rate of about 90% for 1.3 m, while the results of 31-bit gold code show a detection rate of over 95% for 1.4 m. To compare the detection rate with other code at the same distance, the simulation of the 15-bit Kasami code is conducted in the same environment with the 31-bit Gold code. Figure 9 shows that the detection rate of the 15-bit code is approximately 5% lower than that of the 31-bit code. These results show that the long code has the advantage of the ranging and obstacle detection in an interference environment.

### 4.2. Measurement on the Ultrasonic Modem

**Figure 10:**
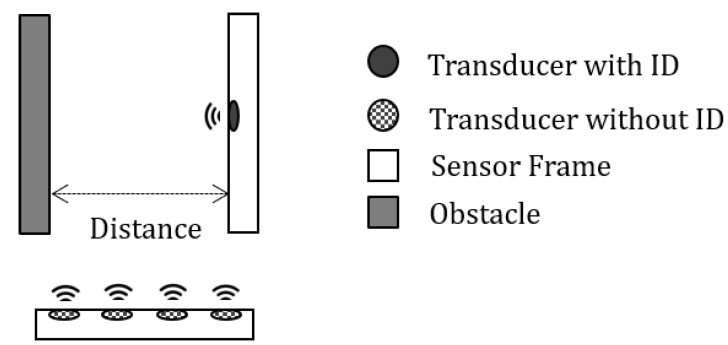
Arrangement of transducers and an obstacle for measurement.

**Figure 11:**
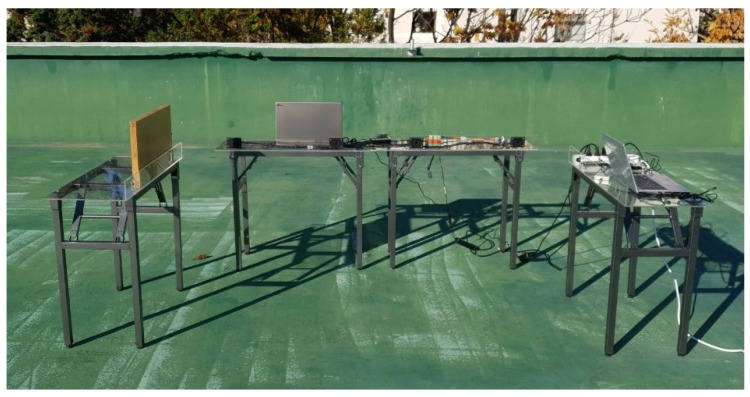
Measurement of the obstacle 2 m way from the transducer.

**Figure 12:**
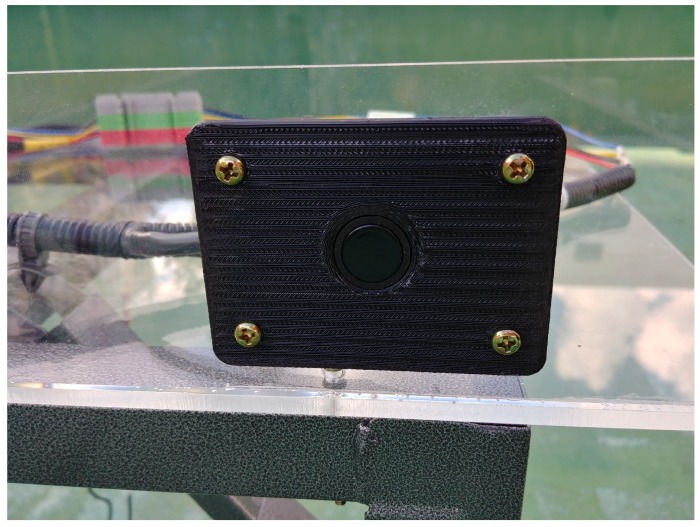
A non-ID transducer to generate interference.

Figure 10 shows the arrange of the transducer and an obstacle for the test, and they are arranged as shown in Figure 11. The obstacle stands at a certain amount of distance from the transducer with an ID. The transducers without ID are placed at the side of the sensor and the obstacle. These sensors are attached to the sensor frame made of an acrylic plate as shown in Figure 12. After finishing the arrangement, the measurement at each distance is conducted while the non-ID transducers generate interference.

The ultrasonic module selects a 7-bit Barker code for a distance of 0.5 m and a 31-bit Gold code for a distance of 2 m. The module repeats the detection 1000 times. Between the ultrasonic modem and an obstacle, there are four transducers to generate interference. The transducers are the rear sensors of the Ssangyong Tivoli, whose transmission routine is the same as the PAS system. It periodically transmits 12 pulses with a 48 kHz carrier frequency. The threshold is 0.3. As a result of repeated measurement, the two detection rate are 97.3% for 0.5 m and 94.5% for 2 m. These results are similar with them of the simulation. The ultrasonic modem in [12] also uses the orthogonal ID, and some outliers occur less than 25 times out of 250 measurements. The detection rate of other work is approximately 90%. Compared to the result of [12], the detection rate in this paper is better in real measurement since the algorithm includes a zero-crossing detector considering the near–far problem.

## 5. Conclusions

In this paper, an algorithm to detect obstacles is implemented. The algorithm ensures that the ultrasonic modem sends a modulated ID with orthogonality instead of a rectangular pulse and detects a reflected wave containing the ID sequence. DQPSK is selected for modulation to satisfy narrow bandwidth and incoherent detection, and a zero-crossing detector is added to solve the near field problem caused by interference. The module determines if it detects the ID by the correlation between the demodulated signal and the ID sequence. It uses 7-bit Barker code or 31-bit gold code according to the distance. Experiment of obstacle detection was conducted with additional sensors generating interference. For measurement, the detection rate of the ultrasonic wave, including each ID, was measured. In the simulation, the detection rate is over 90%, and the results at 1.2 m and 1.3 m show that the long code has the advantage of the higher detection rate at the same distance. In real measurement, the results show 97.3% and 94.5% of detection rate, which are better than the results of other existing work.

## 6. Future Work

The next step to improve detection rate will be to decrease the false alarm rate (FAR), which is the probability of false detection in the absence of reflected waves. If the detection threshold on correlation is too high, the FAR decreases, but the detection probability also decreases. If the threshold is too low, the FAR will increase. Therefore, research to derive adaptive thresholds is needed to reduce FAR while increasing the detection rate. Additionally, the performance test of the ultrasonic modem mounted in the real vehicle should be conducted. The mounted modem verifies the detection of an obstacle as the vehicle moves, and its surroundings are continuously changed. Also, the task to improve the detection rate will be conducted by changing the operation pattern of several sensors, because the driver assistant systems, such as PAS and R-AEB, use four rear sensors on a vehicle.

## Figures and Tables

**Table 1:** Parameters for the simulation.

Parameter	Value
Temperature	20 °C
Atmospheric pressure	101.325 kPa
Relative humidity	40%
Attenuation coefficient	1.46017 dB/m
Reflection coefficient	1
Signal-to-noise ratio	–3, 0, 3 dB
